# 1914. Characteristics of Hospital Onset SARS Cov-2 Infections Before and After the Emergence of The Highly Transmissible Variant B.1.1.529 In a Comprehensive Cancer Center

**DOI:** 10.1093/ofid/ofac492.1541

**Published:** 2022-12-15

**Authors:** Rita Wilson Dib, Amy Spallone, Fareed Khawaja, Jalen Bartek, Sherry Cantu, Tanya Dvorak, Adina Feldman, Hilary McMurry, Leila Nahavandi, Kim Nguyen, Crystal Odom, Amy Hankins, Linda Gravis, Roy F Chemaly

**Affiliations:** University of Texas Health science center, McGovern Medical School, Houston, Texas; University of Texas MD Anderson Cancer Center, Houston, Texas; The University of Texas MD Anderson Cancer Center, Houston, Texas; MD Anderson cancer Center, Houston, Texas; MD Anderson Cancer Center, houston, Texas; MD Anderson cancer Center, Houston, Texas; MD Anderson Cancer center, Houston, Texas; MD Anderson Cancer Center, houston, Texas; MD Anderson Cancer Center, houston, Texas; MD Anderson Cancer center, Houston, Texas; MD Anderson cancer Center, Houston, Texas; MD Anderson cancer Center, Houston, Texas; MD Anderson cancer Center, Houston, Texas; MD Anderson, Houston, Texas

## Abstract

**Background:**

SARS-CoV-2 B.1.1.529 (Omicron) variant was first identified in November 2021 in South Africa and was notable for its increased transmissibility and rapid spread worldwide. In the United States, this variant led to a surge in COVID-19 cases by December 2021. As a result, we experienced a steep rise in cases among patients and employees at our institution starting December 22nd, 2021. Therefore, we compared the incidence and characteristics of hospital-onset COVID-19 (HO-COVID-19) in our cancer patients prior to and during the surge of the Omicron variant.

**Methods:**

We identified HO-COVID-19, as per the CDC definition, from our infection control surveillance database, and additional contact tracing information was reviewed to determine the possible sources of HO-COVID-19. Whole-genome sequencing studies were conducted randomly on nasopharyngeal swabs of patients and employees who had COVID-19 during the study period.

**Results:**

Twenty-six HO-COVID-19 infections were identified from the beginning of the pandemic (February 2020) through February 2022 (Table 1). Only 17 cases occurred over 22 months from the beginning of the pandemic through early December 2021 (Figure 1). These HO-COVID-19 occurred during the 3 COVID-19 surges that were epidemiologically attributed to the variants seen prior to Omicron. Among these 17 patients, 12 (70%) were symptomatic, 9 (53%) had a link to an infected employee, 7 (41%) died during their hospitalization (3 of the deaths were attributable to COVID-19), and 10 (59%) recovered and were discharged. Over 6 weeks (from December 22nd, 2021, through February 1st, 2022), 9 HO-COVID-19 were discovered during the Omicron variant surge (Figure 1). Six (67%) of these patients were symptomatic, 8 (89%) had a link to an infected employee, 2 (22%) died (1 death was attributed to COVID-19 ), and 7 (78%) recovered and were discharged.

**Table 1.**
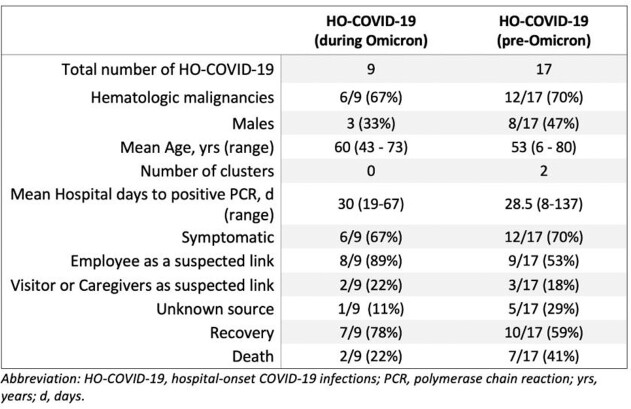
Characteristics and demographics of patients with hospital-onset COVID-19.

**Figure 1.**
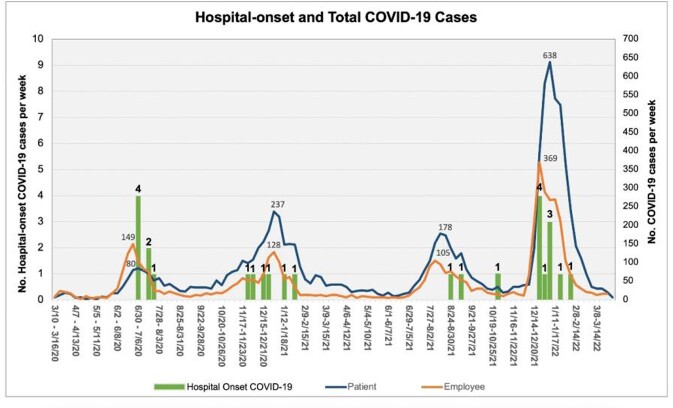
Bar chart of nosocomial COVID-19 cases graphed against line graphs of COVID-19 infections diagnosed weekly among patient and employee at MD Anderson Cancer Center between March 2020 through February 2022

**Conclusion:**

The Omicron variant surge led to marked increases in HO-COVID-19 despite the continuous adoption of enhanced infection control practices, testing on admission, and daily symptoms screening of patients and employees.

**Disclosures:**

**Roy F. Chemaly, MD/MPH**, Karius: Advisor/Consultant|Karius: Grant/Research Support.

**Table 1.** Characteristics and demographics of patients with hospital-onset COVID-19.

Abbreviation: PCR, polymerase chain reaction; HOCI, hospital-onset COVID-19 infections.

